# Repurposing FDA-approved compounds to target JAK2 for colon cancer treatment

**DOI:** 10.1007/s12672-024-01050-9

**Published:** 2024-06-13

**Authors:** Bavya Chandrasekhar, Ravi Gor, Satish Ramalingam, Anuradha Thiagarajan, Honglae Sohn, Thirumurthy Madhavan

**Affiliations:** 1 Computational Biology Laboratory, Department of Genetic Engineering, School of Bioengineering, SRM Institute of Science and Technology, Potheri, Chengalpattu District, Kattankulathur, 603203 Tamilnadu India; 2 Department of Genetic Engineering, School of Bioengineering, SRM Institute of Science and Technology, Potheri, Chengalpattu District, Kattankulathur, 603203 Tamilnadu India; 3 Deparment of Physics with Computer Application, Agurchand Manmull Jain College, Meenambakam, Chennai, Tamilnadu India; 4 Department of Chemistry and Department of Carbon Materials, Chosun University, Gwangju, South Korea

**Keywords:** JAK2, Colorectal cancer, Drug repurposing, Molecular docking, Density functional theory, Molecular dynamic simulation, Cell viability

## Abstract

Colorectal cancer is one of the common cancers worldwide and the second leading cause of cancer-related death. The current treatment has the inherent drawbacks and there is a need of developing a new treatment. Interleukin-6 a pleiotropic cytokine involved in immune regulation and activation of JAK2/STAT3 pathway in colorectal cancer. JAK2/STAT3 signaling pathway functions as a critical regulator of cell growth, differentiation, and immune expression. The abnormality in the JAK2/STAT3 pathway is involved in the tumorigenesis of colon cancer including apoptosis. In this study, we identified novel inhibitors for JAK2 protein by performing virtual screening against FDA-approved compounds. To address the selectivity issue, we implemented cross-docking method followed by DFT calculations to understand the chemical reactivity of the identified compounds. Additionally, molecular dynamics (MD) simulations were performed for the top FDA compounds against JAK2 to understand the molecular interactions and structural stability of the complex over a period of 200 ns. Our results indicated that ergotamine, entrectinib, exatecan, dihydroergotamine, and paritaprevir can be used as alternative drugs for colon cancer. In addition, ergotamine was found to efficiently lower the cell viability with IC_50_ values of 100 µM on colon cancer cell lines. The long-term inhibitory effect of the ergotamine led to a decrease in colony size, and the toxicity properties were studied using hemolysis assay. Our study shows the potential of targeting JAK2 as a novel approach to colon cancer treatment, and demonstrate that ergotamine as a promising effects as an anti-cancer drug.

**Graphical abstract:**

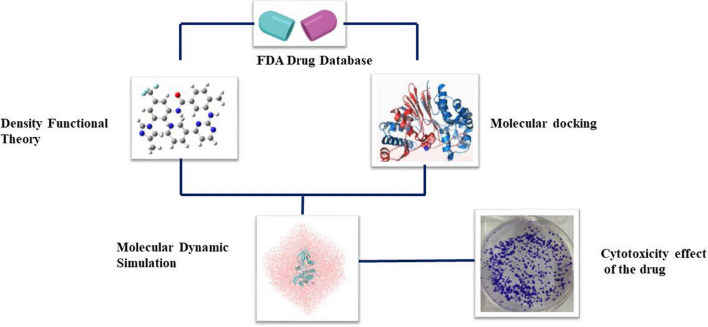

## Introduction

Clinical trial for existing therapeutics for Human Colorectal Cancer (CRC) have a failure rate of approximately 54% due to the severe side effects. CRC is the most prevalent malignancies in the world and ranks as the fourth leading type of cancer according to global cancer statistics between 2002 and 2018 [1–6]. The primary form of CRC can be surgically removed unlike the advanced CRC malignancy, which is associated with a high metastasis rate [7, 8]. Treating local diseases of the colon, such as anorectal issues, Crohn's disease, inflammatory bowel disease, and colonic cancer, as well as the systemic distribution of protein and peptide medications, are receiving a lot of attention. However, the biological mechanism of action for proto-oncogenes and tumor suppressor genes remains poorly understood, yielding fewer targets for therapeutic intervention [9].

The activation of Janus Kinase 2 (JAK2) a non-tyrosine kinase receptor involves in several signaling pathway of pro-inflammatory cytokines which is associated with tumor cachexia. Numerous cytokines, including interferon, interleukin, and growth factors, can attach to type I and type II cell surface receptors, which can activate JAKs protein, and are involved in the molecular mechanisms regulated by IL6/JAK2/STAT3 [10–13]**.** Studies have shown that these matrices foster the survival and growth of tumours, and indicating that bone- marrow derived myeloid cells (BMF) stimulates the carcinogenesis through activation of the IL6/JAK2/STAT3 pathway. This shows that the binding of IL-6 to JAK2/STAT3 induces the activation of the pathway by regulating the cellular proliferation, cell survival, and angiogenesis, contributing to tumorigenesis for CRC. Moreover, the unabated turn-on of oncogene STAT3 results in increased cell generation and impaired cellular apoptosis in CRC [14, 15].

JAK2 /STAT3 pathway inhibition induce cell cycle arrest and apoptosis in CRC. By blocking the activation of JAK2/STAT3 pathway, Bcl-2 is downregulated, Bax is simultaneously upregulated, and the mitochondrial membrane potential is reversed, which starts the apoptotic cascade in CRC [16]. With therapeutic benefits for the inhibitors in the treatment of polycythaemia vera and malignancies, JAK2 enzymes thus represent a significant target for the development of novel drugs [17–23]. Thus, targeting JAK2 will be an intriguing treatment option for CRC [24]**.**

**Fig. 1:**
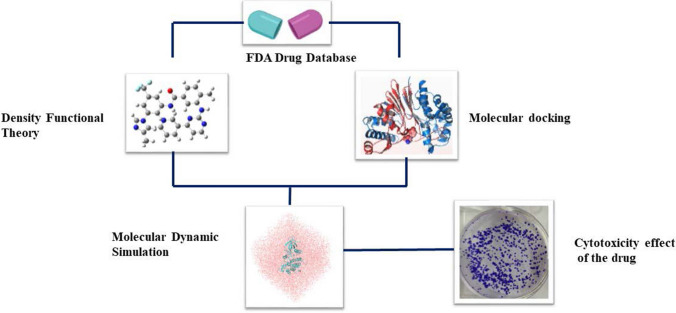
Graphical representation of drug repurposing enhanced by computational methods

Currently approved JAK2 inhibitors include ruxolitinib, baricitinib and fedratinib which target JAK1/JAK2 respectively. Though it has been reported that ruxolitinib and fedratinib have induced thrombocytopenia, anaemia, and mild immune suppression, ruxolitinib possesses off-target interactions that can result in undesired side effects [25]. Drug repurposing or drug repositioning is a process for identifying a new therapeutic drug use that has received prior approval for clinical use [26]. Though, repurposing the FDA-approved drugs as JAK2 inhibitors entails a concerted of in-silico and in vitro/in vivo based methods [27]**.** In this study, FDA-approved compounds were screened against the JAK2 protein active site, which consists of the hinge region, catalytic loop, and DFG motif. The selected FDA compounds were docked with other protein of JAK family, i.e., JAK1, JAK3 and TYK2 by cross-docking method to check the selectivity of the compounds towards the JAK2 protein. The identified FDA compounds were subsequently optimized with the aid of Density Functional Theory (DFT) based Quantum Mechanics (QM) method using B3LYP/6311++ G (2d, p) levels to predict the electronic structure and reactivity. MD was performed to understand structural stability, binding interaction and residual flexibility of the selected ligand complexes. Finally, ergotamine was selected for in vitro study, and the compound efficacy, sensitivity and toxicity were assessed using a cell viability, colony formation and hemolysis assays using HCT 116 colon cancer cell lines represented in Fig. 1.

## Materials and methods

### Protein selection and preparation

The PDB structure of the JAK2 protein (PDB ID: 4IVA) bound to the co-crystal ligand was retrieved from RCSB (http://www.rcsb.org/) [28]. The protein preparation involving the removal of crystallographic water molecules and the co-crystallized ligand 1J5 was performed using PyMol software (http://www.pymol.org/) [29]. Further, the protein preparation entitled adding of polar hydrogens; Kollman and Gasteiger charges and energy minimization was performed in the Auto dock tool. The resulting structure was saved in a pdbqt format to perform molecular docking.

### Selection and preparation of ligand for molecular docking

The FDA drug database consisting of drugs approved for clinical use was downloaded from in sdf files in order to be used as ligands for docking. The database provides the details in depth about the bioactivity, characteristic and safety. Open Babel software is used for virtual screening to generate 3D coordinates from 2D representations to prepare the FDA compounds for docking algorithms. The converted 3D structures of compounds were imported as pdbqt format, and utilized in the Autodock Vina software installed in HPCC, SRM Institute of Science and Technology, Chennai.

### Docking site for JAK2 protein

Various clinical-stage JAK inhibitors focused on targeting the kinase domain ATP binding pocket for inhibiting [30]. The ATP binding residues in the protein JAK2 were examined and the area around the co-crystal ligand site around 4Å are identified as the most optimal. There are different set of residues found in the ATP pocket that includes Active site residues Leu855, Gly856, Lys857, Val863, Glu930, Tyr 931, Leu932, and Met929 which includes catalytic loop, hinge region, DFG motif where the DFG motif controls protein kinase catalysis and aids in ATP binding, and gatekeeper residues for ligand docking which they have been identified through previous literature studied.

### Virtual screening and molecular docking methods

Virtual screening (VS) is the process of identying potent compounds from large chemcial or natural database by ligand and structure based pharmacophore modeling, and molecular docking techniques. Herein, we performed structure-based VS, where each FDA compound is docked at the JAK2 binding site and their corresponding binding energy is calculated by Autodock Vina and Glide docking algorthims [31].

The Lamarckian genetic algorithm conformation search was used to perform molecular docking with default parameters with Autodock Vina built in AutoDock tool 1.5.7. The partial receptor flexibility was given only to the specific active site residues and the complete conformational flexibility were given to the ligand including rotatable bonds and lengths. The docking poses preferred was determined with the similarity with co-crystal ligand serve as the initial process in docking method [32]. The site specific molecular docking was performed were the FDA approved compounds were docked at JAK2 ATP binding domain. The top ten compounds were selected based on the scoring values, binding pose compared with the existing co-crysal conformation, and further these compounds were validated with Glide docking alogrithms.

#### Glide docking method

The selected FDA compounds were docked with the site specific ATP binding domain was performed by Glide XP models using the Glide package in Schrondinger. The protein was prepared using protein preparation wizard by applying default paramteres which includes hydrogen bond optimization, filling the missing atoms and assinging bond orders. In the receptor grid generation, the center of the grid box was defined on the centroid of co-crystallized ligand and the default paramters was used to calculate the volume of active site region. The docking was performed using Extra Precision (XP) modes and the receptor was held rigid and the ligands were free to move. Glide score is calculated which a combination of hydrophobic,hydrophilic, van der Waals energy, polar interactions and freezing rotatable bonds and Glide XP score reduces the false positive results and was considered for the selection and further analysis. The interaction between the protein and ligand were visualized.

### Cross-docking methods

Cross-docking was performed to check the selectivity of the identified FDA compounds with other closely related JAK protein such as JAK1, JAK3 and TYK2. The crystal structure of JAK1 (PDB ID: 6N7D), JAK3 (PDB Id: 5TOZ) and TYK2 (PDB ID: 4GJ2) was retrieved from the Protein Data Bank website. The searching and scoring function parameters were maintained similar to those of JAK2 protein. The top selected ligands from molecular docking were docked against JAK1, JAK3 and TYK2 using Auto dock Vina 1.5.7 software to identify the selective inhibitor [33].

### Conceptual density functional theory

A quantum mechanical method for calculating the characteristics of atomic systems is conceptual density functional theory (DFT). Hohenberg–Kohn theorem work has given rise to density functional theory, which makes use of spatially dependent electron density functions to understand the properties of different electron systems. C-DFT, which is a subfield of DFT, was used to study the chemical properties of the molecule-supported electron density concept. [34]. C-DFT a sub-field of DFT was performed to analyses the chemical behavior of the molecule-based on electron density concept. Ten molecular descriptors were investigated, including reactivity descriptors and their derivatives, to explain the orbital features. Total energy, Global softness (σ; in eV-1), Lowest Unoccupied Molecular Orbital (LUMO), Highest Occupied Molecular Orbital (HOMO), Dipole moment, Electronegativity (χ), Energy gap, Chemical potential (μ; in Ev), and Electrophilicity index (ψ; in Ev-1) are all included. [35–38]. The descriptors are calculated based on Fukui's molecular orbital theory. The ability of a molecule to receive and donate electrons depends upon EHOMO and ELUMO descriptors. The total polarity of the system was measured by molecular dipole moment. The descriptors _, σ, χ, μ, and ψ, which represent electron sharing or transfer from the HOMO to LUMO, are obtained from EHOMO and ELUMO. Using Gaussian 16 software’s B3LYP function and 6–31G (d) basis set, the chosen ligands were optimized [39].

### Molecular dynamic simulation

The MD Simulation was carried out Desmond tool of using Schrodinger Drug Design Suite. The MD simulation were performed to investigate the stability in holo-form ( protein- ligand complex) and apo form (only protein) with five short listed compounds from molecular docking and DFT. Simulation of protein JAK2 and complexes ergotamine, entrectinib, exatecan, paritaprevir and dihydroergotamine were carried out for 200 ns. Simple point charge (SPC) with OPLS 2005 force field were used in the simulation. To equilibrate, the counter ions Na^+^/Cl^−^ were added to balance charges in the system. The system was energy-minimized and the system was equilibrated at the temperature 300 k and 1 atmosphere pressure. The equilibrated system was used to finally perform the unrestrained MD simulation for 200 ns. The final MD trajectories for each complexes was studied and the stability of the protein–ligand complex was studied by RMSD (Root mean square deviation), RMSF (Root mean square fluctuations), Protein – ligand interactions and contacts with amino acids were studied [40].

### Hemolysis

Estimating the hemolysis is a frequently used method to understand the cytotoxic level of the inhibitor. To perform, we collected fresh human blood to which EDTA was added as an anticoagulant agent. The red blood cells (RBC) were isolated by adding 4 ml of phosphate-buffered saline (PBS) to the 2 ml sample was added and centrifuges at 10,000 ˟ g for 5 min to from the blood. 10 ml of PBS was added each time to the RBC’s and washed for five times and diluted adding 20 ml of PBS. For the evaluation, 0.1 ml of the diluted sample was taken and IC50 concentration of the drug ergotamine was added, to the positive control distilled water was added and PBS was added as the negative control. The tubes were incubated at 37 ºC for 24 h and 48 h in incubator. After 24 h the samples were centrifuges again at 10,000 ×*g* for 5 min. To measure the absorbance at 590 nm. 100 µl from the supernatant was transferred into 96 well plate. Hemolytic ratio was quantified using the formula [41–43]

$${\text{Hemolytic ratio}} = \frac{{{\text{OD }}\left( {{\text{test}}} \right) \, {-}{\text{ OD }}\left( {\text{negative control}} \right)}}{{{\text{OD }}\left( {\text{positive control}} \right) \, {-}{\text{ OD }}\left( {\text{negative control}} \right)}} \times 100$$

### Cell viability assay

The NCCS in Pune provided the colorectal cancer cell line HCT-116, which was cultivated in Dulbecco modified Eagle’s medium (DMEM) supplemented with 1% antibiotics and 10% fetal bovine serum (Gibco). The cultured cells were kept at 37 ºC with 5% CO_2_ in an incubator that was humidified. Viability of cells was assessed using the MTT test 3-(4,5-dimethylthiazol-2-yl)-2,5-diphenyltetrazolium bromide to ascertain the impact of ergotamine. In a 96-well plate, 5000 cells were seeded per well. The cells were then treated with several dosages of ergotamine (25, 50, 75, 100, and 200 µM) and incubated at 37 ºC with 5% CO_2_. To the each well MTT was added and incubated at 48 h, and the plates were then incubated for 4 h at 37 ºC. Afterwards, 100 μl of DMSO was used to solubilize the formazan crystals. Measurements of absorbance were made at 570 nm using a microplate reader and the percentage cell viability graph was plotted [43, 44].

### Colony formation assay

HCT116 cells with density of 500 cells/well were added to 6 well plate and then incubated overnight (37 ºC). The medium was then removed and IC50 value ergotamine-containing medium was added to incubate for 48 h. After 48 h, the drug-containing medium was removed. Fresh medium was then added and continued to incubate for 10 days (10–48 h). After 10 days, the medium was again removed and cells were washed with PBS (PBS) (10% formalin). Stained cells were washed twice with PBS (10% ethanol) (1% crystal violet). Colonies were counted in treated wells (treated) and untreated control [45].

## Results

### Molecular docking

**Fig. 2:**
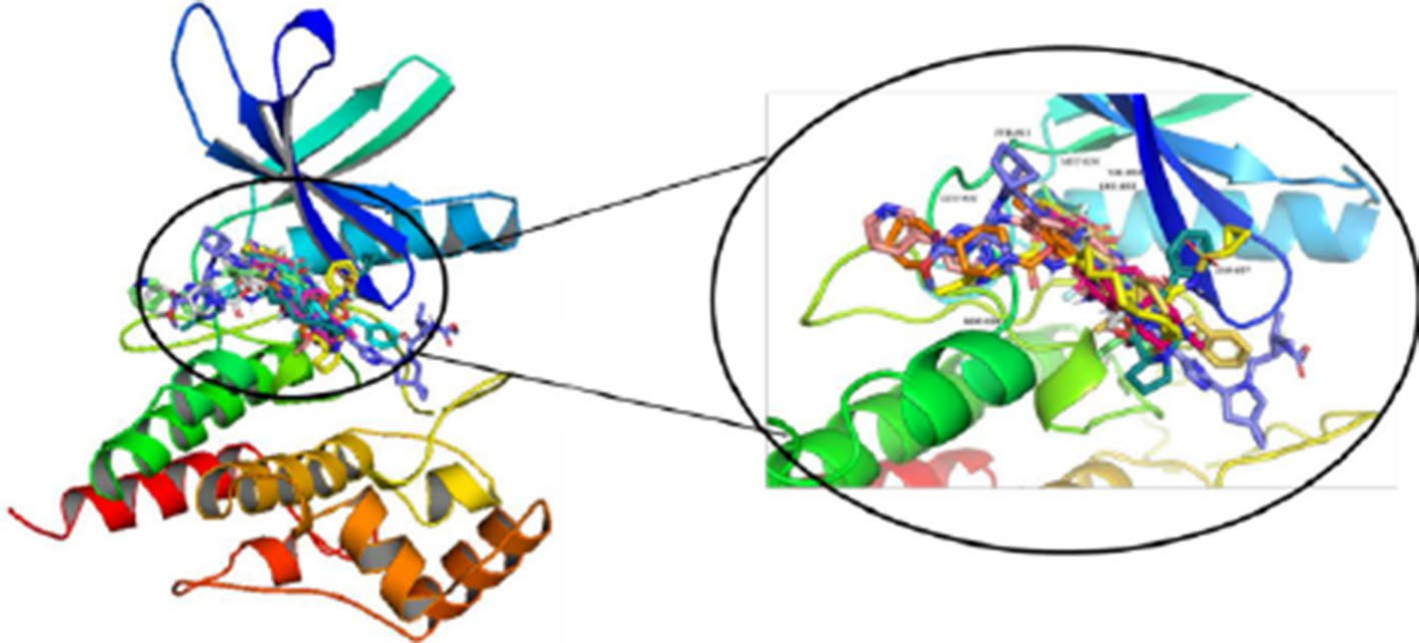
JAK2 (PDB ID: 4IVA) structure with the interactions of the FDA compounds present inside the ATP binding site after molecular docking

The FDA-approved compounds were docked at the active site of the JAK2 protein using Autodock Vina. Based on the binding interaction and similar binding pose of the co-crystal confirmation top ten compounds were chosen. The selected compounds were validated by performing the site specific docking with Glide docking algorithms and Auto Dock tools. The selected compounds include Erogtamine, Entrectinib, Exatecan, Dihydroergotamine, Paritaprevir, Irinotecan, Ledipasvir, Lumacaftor, Nilotinib and Benazeprilat which showed higher binding affinity on both docking modules were tabulated in Table 1. On analyzing the interactions each amino acid residue within 4 Å distance from the ligand was studied to know the presence of hydrogen and hydrophobic bond interaction. The residues Glu930, and Leu932 are the key amino acids in the active site of the protein. These ligands show one or more interactions with Glu930, Lys857, Leu855, Leu932, Ser936, Asp994, Lyn857 and hydrophobic interaction Leu855, Glu856, Val863, Ala880, Tyr931, Ser936, Asp 939, Arg981, Leu983 account for the stability of the compound in the pocket are depicted in Table 2 and Fig. 2. The top ten compounds with the higher binding affinity and interaction with the key amino acids, were selected for the further study. The 2D structure of the identified FDA compounds are given in Table 3.

**Table 1:** Binding affinity of the FDA compounds with JAK2(PDB ID 4IVA) protein, the H–bond interaction, and Hydrophobic interactions with amino acid residues in the binding site

FDA Drugs	Docking score (kcal/mol)	Glide XP Score (kcal/mol)	H-bond interactions	Hydrophobic interactions
Ergotamine	− 11.4	− 11.1	Glu930,Lys857	Leu855, Leu932, Val863, Val911, Ser936, Gly935, Gly993, Met929
Entrectinib	− 11.4	− 10.9	Leu855,Leu932,Ser936	
Exatecan	− 11.3	− 10.9	Asp994,Lyn857	
Dihydroergotamine	− 11.0	− 10.7	Glu930	
Paritaprevir	− 10.8	− 10.5	Asp939,Tyr934	
Irinotecan	− 10.7	− 10.3	Leu932	
Ledipsavir	− 10.5	− 10.3	Leu855	
Lumacaftor	− 10.4	− 10.3	Leu932	
Nilotinib	− 10.3	− 10.4	Ser862	
Benazeprilat	− 10.3	− 10.3	Lys857,Ser936

**Table 2:** 2D interactions of the lead compound and all of non-bonding interactions

FDA drugs	Interactions	FDA drugs	Interactions
Ergotamine		Nilotinib	
Exatecan		Entrectinib	
Dihydro ergotamine		Paritaprevir	
Lumacafactor		Irinotecan	
Ledipsavir		Benazeprilat

**Table 3:** Chemical structures of lead ligands after screening with JAK2

FDA compounds	Structure	FDA	Structure
Entrectinib		Pariteprevir	
Dihydroergotamine		Nilotinib	
Ergotamine		Ledipsavir	
Exatecan		Benazeprilat	
Irinotecan		Lumacaftor	

### Cross-docking

To achieve the intra-family selectivity cross-docking was performed for the top ten selected compounds. Cross docking was performed with JAK1(6N7D), JAK3 (5TOZ) and TYK2 (4GJ2) structures with the selected FDA compounds which showed higher binding affinity in the molecular docking study. When compared to JAK1, JAK3 and TYK2, JAK2 shows better binding affinity. JAK2 (> − 11.0 kcal/mol) has the highest binding energy when compared to JAK1 (> − 8.9 kcal/mol), JAK3 (> − 8.9 kcal/mol) and TYK2 (> 9.0 kcal/mol). The binding affinity of all JAK proteins is represented in Table 4, and the selected FDA compounds were studied further.

**Table 4:** Cross-docking results of selected JAK2 leads against JAK1, JAK3 and TYK2

FDA Drugs	Binding Affinity kcal/Mol			
	JAK2 (PDB ID:4IVA)	JAK1 (PDB ID:6N7D)	JAK3 (PDB ID:5TOZ)	TYK2(PDB ID: 4GJ2)
Ergotamine	− 11.4	− 8.9	− 8.9	− 9.0
Entrectinib	− 11.3	− 8.7	− 8.9	− 8.9
Exatecan	− 11.3	− 8.2	− 8.5	− 8.9
Dihydroergotamine	− 11	− 8.4	− 8.4	− 9.0
Paritaprevir	− 10.8	− 8.3	− 10.3	− 8.7
Nilotinib	− 10.8	− 7.5	− 7.6	− 9.1
Ledipsavir	− 10.8	− 7	− 7.4	− 9.5
Lumacaftor	− 10.8	− 7.3	− 7.6	− 8.5
Irinotecan	− 10.7	− 7.7	− 8.6	− 8.9
Benazeprilat	− 10.7	− 5.3	− 5.8	− 7.2

### C- DFT

The orbital energy of the comounds was investigated using C-DFT [46]. The molecular descriptors ∆E, µ, ω, χ, η, σ of the compounds were analysed based on HOMO energy and LUMO energy are calculated and mentioned in Table 5. Results indicated that ergotamine showed the least energy gap of ∆E = 0.17 e V, followed by exatecan ∆E = 0.56 e V and dihydroergotamine ∆E = 0.60 e V. The energy needed to perform the transition of the molecule from the lower orbital to the higher orbital is represented by the energy difference (E) = E (LUMO)—E (HOMO) frontiers orbital, [47]. The maximum D_p_ was shown by ergotamine [D_p_ = 12.9 Debye units]. Chemical reactivity has a direct relationship with the molecular dipole moment. Calculating the derived descriptors, the most electronegative compounds was fount to be dihydroergotamine [χ = 5.24] and paritaprevir [χ = 4.25]. The global softness and absolute hardness were the criteria for the stability of the system and they found to be in acceptable range respectively. Chemical potential is the negative value of electronegativity, which also indicates high chemical reactivity. Therefore, dihydroergotamine and paritaprevir exihibited high chemical potential. High electrophilicity index was seen for entrectinib [4.6] and paritaprevir [3.83] suggests to have likeliness to accept the electron. These calculated statistical values of the molecular descriptor are tabulated in Table 6. The higher the dipole moment, lower electronegativity and smaller energy gap are the important parameters for the efficient inhibitory effects of the selected compounds.

**Table 5:**
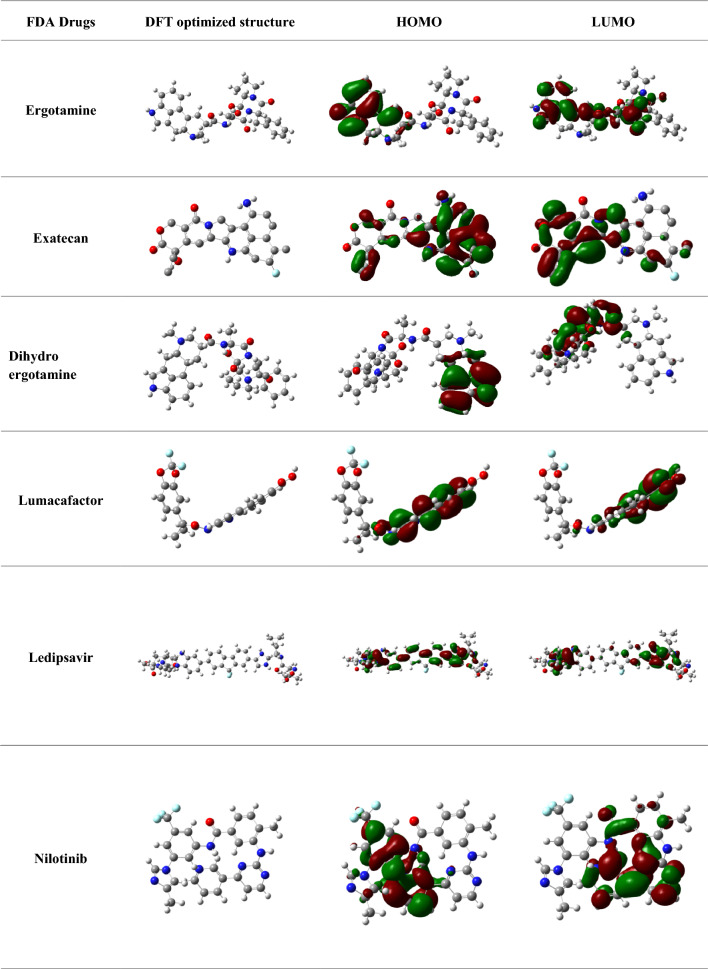

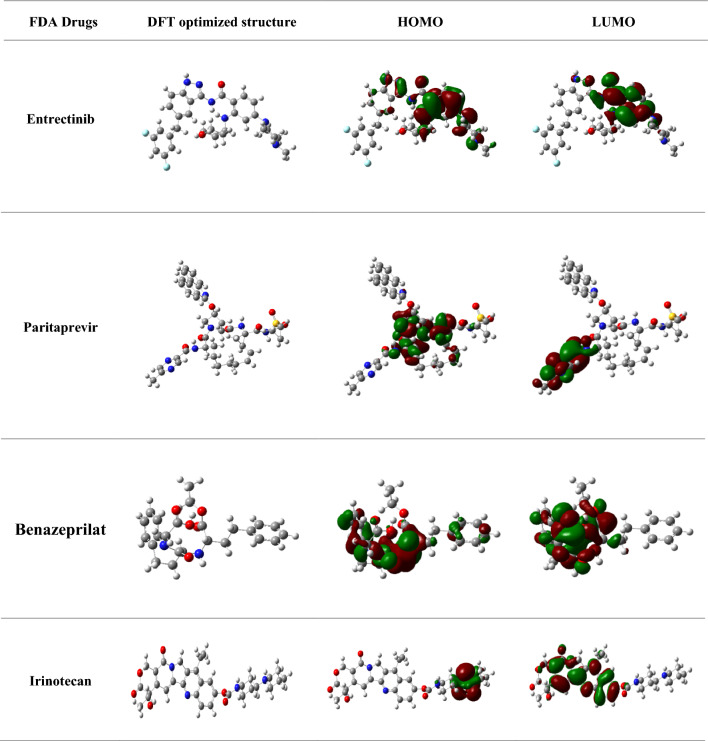
Statistical results of DFT-based descriptors and Electron density maps of HOMO and LUMO of selected FDA compounds

**Table 6:** Statistics of DFT-based molecular descriptors of selected FDA compounds against JAK2

FDA compounds	Total Energy (E γ)	Molecular dipole moment (Debye)	E_HOMO_	E_LUMO_	HOMO/ LUMO Gap	Absolute Hardness	Global Softness	Electronegativity	Chemical potential	Electrophilicity index (ω)
	(in eV)				(ΔE)	(η)	(σ)	(χ)	(μ)	
Ergotamine	− 52,473.3	12.9	− 5.43	− 1.0	0.17	2.2	0.22	− 3.22	3.22	− 3.56
Entrectinib	− 51,163.6	5.7	− 5.97	− 1.25	4.71	2.35	0.21	− 3.61	3.61	− 4.6
Exatecan	− 156,498.3	7.0	− 0.14	− 0.71	0.56	0.28	1.75	− 0.430	0.43	0.326
Dihydroergotamine	− 52,507.0	6.4	− 5.55	− 4.94	0.61	0.30	1.63	− 5.24	5.24	− 0.79
Paritaprevir	− 77,754.3	11.5	− 6.05	− 2.45	3.60	1.80	0.27	− 4.25	4.25	− 3.83
Nilotinib	− 50,000.1	7.7	− 0.22	− 0.05	4.43	0.08	5.88	− 0.135	0.165	0.10
Ledipsavir	− 2.989.0	10.3	− 2.01	− 0.33	1.68	0.84	0.59	− 1.17	1.17	− 1.31
Lumacafactor	− 4.3823	4.0	− 0.13	− 0.09	0.44	0.02	2.5	− 0.11	0.11	0.30
Irinotecan	− 144,446.1	8.5	− 6.04	− 2.25	3.79	1.89	2.6	− 4.14	4.14	− 2.92
Benazeprilat	− 38,559.3	2.67	− 6.1	− 1.01	− 5.08	− 2.54	− 0.19	− 3.55	3.55	− 2.52

### Molecular dynamic simulation

A 200 ns MD of JAK2 was performed to understand the receptor stability, ligand interaction and conformational flexibility of complexes and apo form of JAK2 protein. The fluctuations in the simulation of protein–ligand complex were explored in the SPC system. RMSD, RMSF plot, and H-bond were used to estimate the stability and structural comparison of the protein and complexes.

**Fig. 3:**
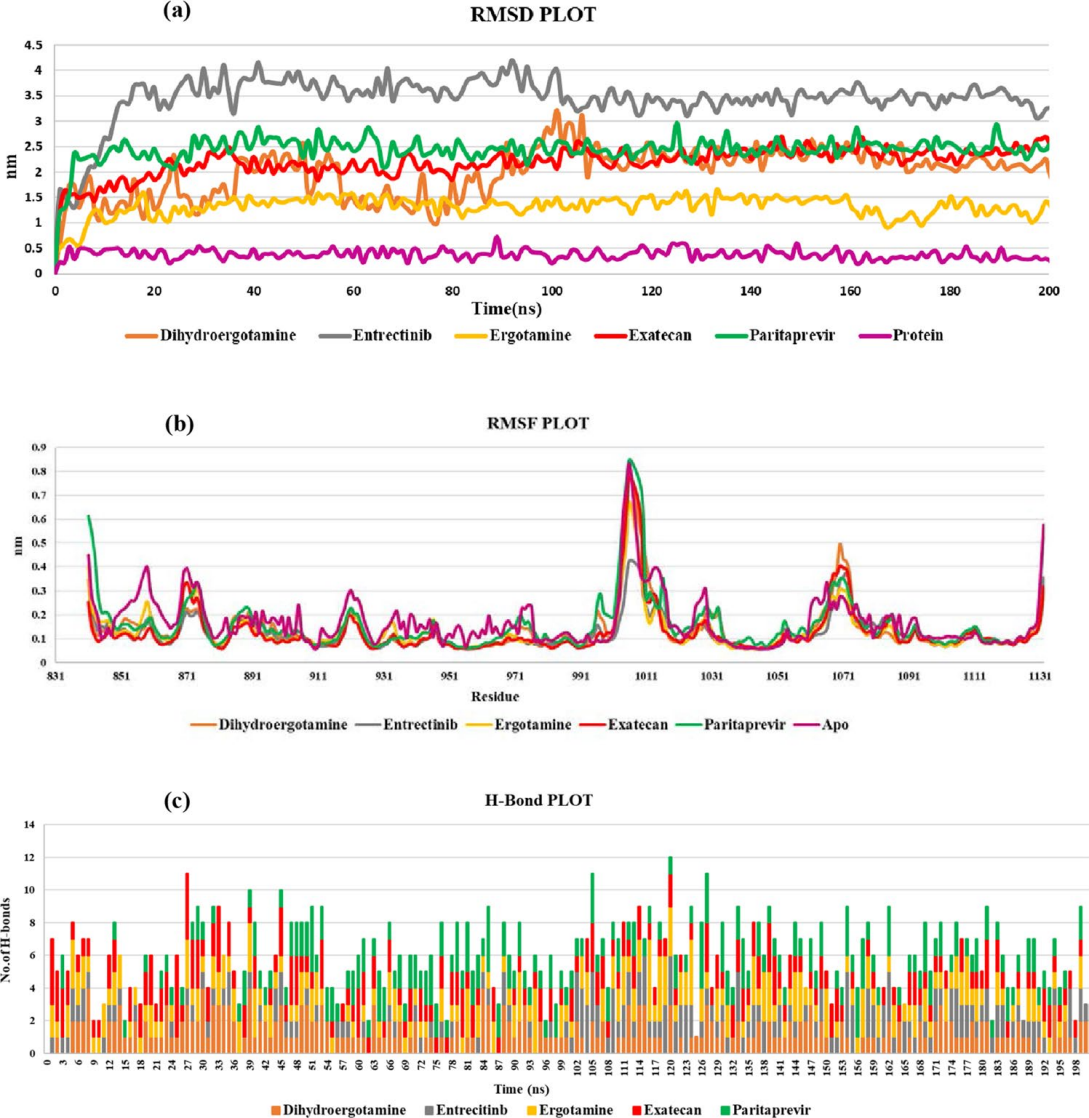
**a** The RMSD values of backbone atom of JAK2 (PDB ID: 4IVA) after binding with the FDA compounds over a period of 200 ns. **b** The RMSF Plot of JAK2 structure after binding with the FDA compounds over a period of 200 ns. **c **H-bonds formed by FDA compounds with the JAK2 protein over 200ns

The RMSD (Root Mean Square Deviation) of residues of Cα atoms within 4 Å of the ligand in an JAK2 active site pocket was demonstrated in comparison to the initial structures. The RMSD in MD Simulation is to analyze the degree of constancy for the selected compounds among the binding site of JAK2 protein. The RMSD for the complexes was calculated for 200 ns. In the Fig. 3a, RMSD plot shows that the apo protein has undergone minor deviations till 200 ns of simulation up to ~ 0.5Å, and the deviation has been reduced from ~ 70 ns confirming the stability of the protein–ligand complex less than 4Å. In the case JAK2–Dihydroergotamine the initial deviations were first found to be at first 100 ns up to ~ 3Å and after that the minor deviations were found up to ~ 2.5Å throughout the simulation. Complex JAK2- Entrectinib the deviations were seen from 20–100 ns up to ~ 4Å and later the minor deviations were found from 120–180 ns up to ~ 3.5Å and stayed stable till 200 ns. For the complex JAK2- Ergotamine minor deviations less than ~ 1.5Å throughout 200 ns. In the case of JAK2- Exatecan the deviations were found to be 20–80 ns up to ~ 2Å and deviations stayed stable till 200 ns. JAK2—Paritaprevir complexes the deviations were found from 20 to 120 ns up to ~ 2.5Å and later the deviations were found to be minor throughout the simulation up to ~ 2.5Å. The results indicated that the selected FDA compounds shows smaller fluctuations and observed high stability during simulation.

The RMSF (Root mean square fluctuation) method was performed to understand the JAK2 protein–ligand flexibility, dynamic behaviour, and FDA compound binding conformation over a period of 200 ns. In the Fig. 3b, RMSF backbone atoms were monitored to check the strong binding interactions of these compounds in the ATP site of the JAK2. The results showed that the RMSF of the JAK2 showed a similar pattern of fluctuation; the observed dynamic fluctuations were assigned to the domains that they found in the inactive site regions or near the C-terminals and N-terminals, such as the domains around residues ASN874, ARG922 and ASP1068 for JAK2 complexes. Other residues formed hydrophobic interactions with Leu855, Glu856, Val863, Ala880, Tyr930, Glu934, Leu983 JAK2. The important and selective residues Leu932, and Glu930 formed favourable interactions that are important for inhibitor binding.

**Fig. 4:**
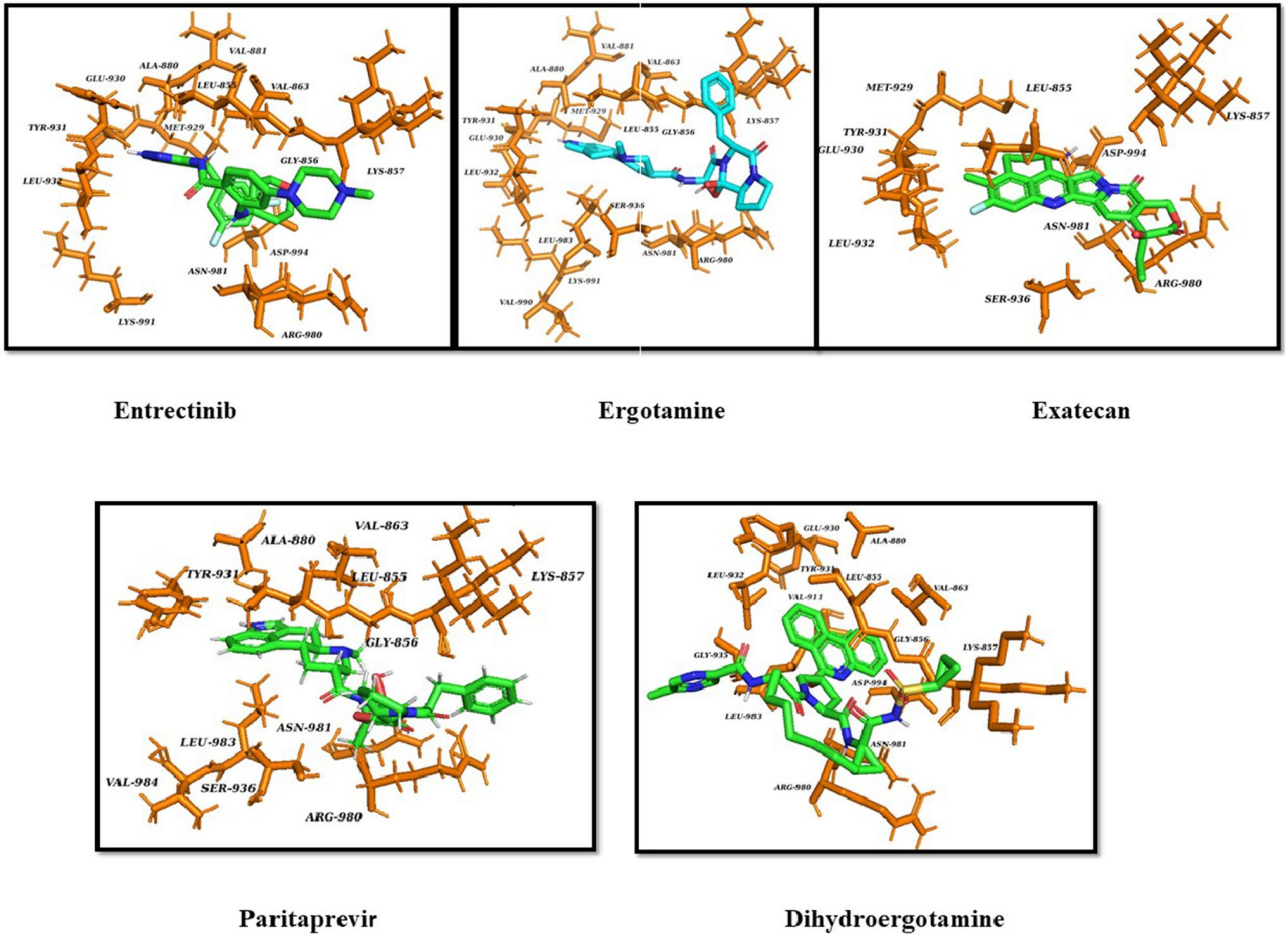
The proposed binding confirmation of FDA compounds present in the ATP binding pocket of JAK2 after 200 ns

The significant part of the protein–ligand interaction in MD simulation illustrates the changes in the binding mode during the simulation. The H-bond is analysed to know the stability of the protein–ligand complex and it plays a vital in adsorption and precision of drug during the process of drug designing. The bonding pattern of JAK2 protein with the complexes during the 200 ns simulation is plotted in Fig. 3c. The trajectories in the MD simulation were analysed to understand the hydrogen bonds formed between protein and ligand complex. The hydrogen bonds were 1–4 H—bonds for ergotamine and exatecan and 1–4 hydrogen bonds were seen between the protein and Entrectinib, for the protein–Paritaprevir complex there were about 1–3 H-bonds and for Dihydroergotamine it was around 1–4 H bonds are formed. The bonding parameters demonstarted the complxes bound to JAK2 protein tightly and effectively. These results showed that the selected compounds are stable and expected the potential therapeutic compounds of JAK2 against CRC. In the interacting residues of the protein–ligand complexes before and after the simulation were analyzed and represented in Table 7. The binding conformation of the drugs before and after simulation is displayed in Fig. 4. The conformations were stable in the ATP pocket, which prove the realibility of the docking study. Further, these analysis suggests that the identified FDA compounds are selective and potent and can be studied for in vitro validations.

**Table 7:** Protein–ligand interactions of JAK2 and the FDA compounds before and during molecular dynamic simulation

S.no	FDA Drugs	During Molecular dynamic simulation			
		50 ns	100 ns	150 ns	200 ns
1	Ergotamine	Leu932	Leu932	Leu932	Lys857,Leu932
2	Entrectinib	Leu932,Ser936	Ser936	Ser936	Leu932,Ser936
3	Exatecan	Asp994	Leu855	Ser936	Asp994.Arg980
4	Dihydroergotamine	Ser1039	Ser1039	Ser1039	Glu890
5	Paritaprevir	Lys857	Lys857	Lys857	Lys857

### Ergotamine inhibits colon cancer cell viability

**Fig. 5:**
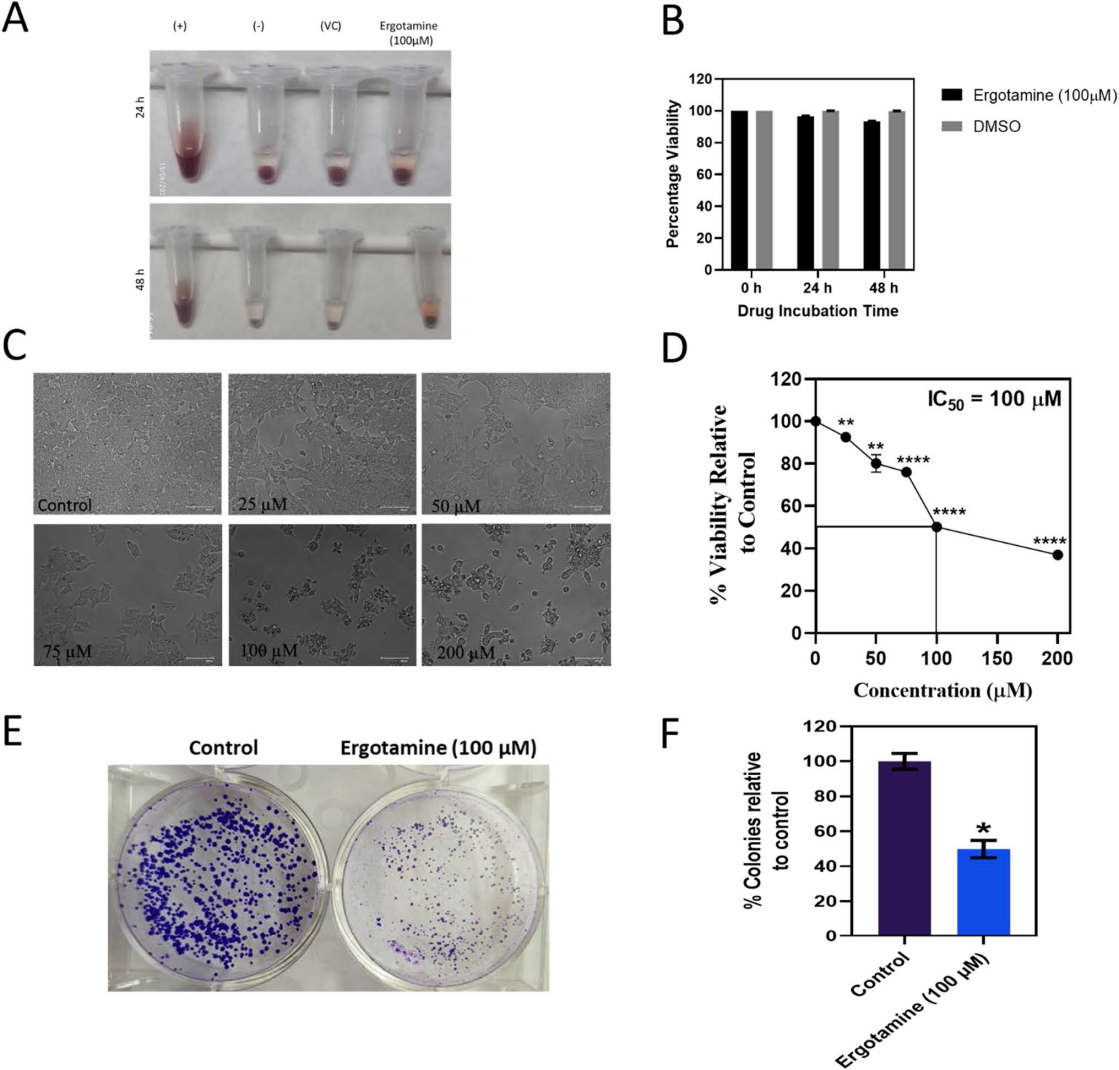
Cytotoxic effect of ergotamine: **A** Representative image showing hemolysis in treated and untreated controls. **B** Graph representing the percentage red blood cell viability at 24 and 48 h. **C** Morphology of colorectal cells post-treatment with ergotamine and untreated control. **D** Reduced colorectal cell viability post-treatment with ergotamine, and IC50 were calculated and found to be 100 µM. **E** Representative image of colonies stained with 1% crystal violet. **F** Graph representing percentage of colonies relative to untreated control wells. The graph represents mean ± SEM, ^*^P < 0.05, ^**^P < 0.01, ^****^P < 0.0001

We performed hemolysis, MTT cell viability assay and colony formation assay to understand the cytotoxic effect of ergotamine. A wide range of ergotamine concentrations were used to access the minimum inhibitory concentration required to kill 50 percentage of the cell population (IC50), concentrations were ranging from 25, 50, 75, 100, and 200 µM. We have observed dose dependent decrease in colon cancer cell viability and IC50 concentration were found to be 100 µM from the graph (Fig. 5C and D). We found that there was minimum hemolysis when red blood cells (RBCs) were treated with IC50 concentration of ergotamine when compared with untreated control (Fig. 5A and B). To evaluate the long term effect of ergotamine we have performed colony formation assay, here we observed significant decrease in number and size of the colonies when compared to the untreated control wells (Fig. 5E and F). Our results show that ergotamine is effective in inhibiting the viability of colon cancer cells whereas not affecting the normal RBCs.

## Discussion

Colon cancer is recognized as one of the prevalent cancers globally and the occurrence is influence by different factors including age, family, lifestyle and diet. JAK2 signaling has an important role in the solid tumors like colon cancer, lung cancer, prostate etc. The recent literature suggested that inhibition of JAK2 leads to apoptosis and cell cycle arrest in colon cancer. To target JAK2, we performed structure based virtual screening method that includes, molecular docking, and MD simulation using drug repurposing method.

FDA compounds were docked at the JAK2 protein ATP binding site, shows that Leu932, Glu930, Asp 994, Leu855, Val863, Ala880, Met929 and Leu983 were the interacting residues between the protein and ligands. Obtaining a reasonable level of intra-family selectivity is a key goal. The selectivity of the compounds against JAK2 protein showed (> − 11.4 kcal/mol) compared with JAK1, JAK3 and TYK2. The protein–ligand interactions were analyzed, the binding poses and interactions with the residues were studied and displayed to get a better understanding of these interactions governing the binding of the FDA compounds to the JAK2 protein. The top ten compounds which showed the highest binding scores, were studied further. DFT calculations was used to analyzed the chemical reactivity, smaller energy gaps, lower electronegativity, dipole moment, softness, hardness and electrophilicity index were studied. The MD simulation was performed for the selected top five FDA compounds to investigate the binding conformation with the JAK2 protein. RMSD was studied using the apo- protein as a reference for the complexes and the change in each amino acid residue is understood by RMSF. The correlation between the apo and complexes suggests a similarity in the structural dynamics. The stability of the complexes is influenced by the hydrogen bonds between the complexes and the interaction was observed throughout the 200 ns simulation for all the compounds.

The drug Exatecan belonging to topoisomerase I inhibitory and anti-cancer agent has been in trials for treating Leukemia, lymphoma, Lung cancer and liver cancer [48]. The antitumor properties of Exatecan is investigated in various model system in in vitro and in vivo [49]. Entrectinib belonging to antineoplastic used for the treatment of metastatic non-small cell lung cancer. The drug Paritaprevir is used as anti-viral drug to treat chronic Hepatitis-C infection. The mechanism of action is as a protease inhibitor and P-glycoprotein inhibitor [50]. Dihydroergotamine belonging to ergot alkaloids is a derivate of Ergotamine [51]. As the purpose of this study is to identify the novel clinical usage of existing drug for a different indication, we are more focusing on Ergotamine [52]. Ergotamine belonging to the class of medications called ergot alkaloids. It prevents the blood vessels in head from expanding which causes headaches. The inhibitory effects of ergotamine on colon cancer cells lines HCT116 was studied through colony formation assay and cell viability assay. The toxicity of the drug was studied in colon cell lines as well normal human cell lines. By using the drug repositioning strategy, ergotamine can be suggested as an anti-cancer agent. Further, in vitro and in vivo study is recommended to understand their anti-cancer activity.

## Conclusion

The important aspect of drug repurposing is to decrease the time of drug development and availability of the among the patients. Molecular docking study were used to identify potent and selective FDA compounds against JAK2 protein. From the docking study, the interactions and the binding affinity were briefly studied. The FDA compounds Ergotamine, Entrectinib, Exatecan, Dihydroergotamine and Paritaprevir can be used a promising inhibitor against JAK2 protein of colon cancer. Ergotamine, the selected FDA compound was further used to access its cyto-toxicity effects on normal red blood cells RBCs and HCT116 colon cancer cells. We have observed Ergotamine is effective in inhibiting colon cancer cell viability whereas there was no significant hemolysis over IC_50_ concentration of ergotamine and it has also shown, the reduce in number and size of colonies in ergotamine treated wells when compared to untreated control. Further, in vitro and in vivo experiments have to be performed to understand the efficacy and safety of these drugs.
